# Comparing three diagnostic algorithms of posttraumatic stress in young children exposed to accidental trauma: an exploratory study

**DOI:** 10.1186/s13034-015-0046-7

**Published:** 2015-05-13

**Authors:** Maj R. Gigengack, Els P. M. van Meijel, Eva Alisic, Ramón J. L. Lindauer

**Affiliations:** Department of Child and Adolescent Psychiatry, Academic Medical Center, University of Amsterdam, Meibergdreef 5, 1105 AZ Amsterdam, The Netherlands; de Bascule, Academic Center for Child and Adolescent Psychiatry, Meibergdreef 5, 1105 AZ Amsterdam, The Netherlands; Monash Injury Research Institute, Monash University, Building 70, 21 Alliance Lane-Monash University, Clayton Campus, Melbourne, VIC 3800 Australia

**Keywords:** Posttraumatic stress disorder, Young children, Preschool, Accident, DSM-5

## Abstract

**Background:**

Both the DSM-5 algorithm for posttraumatic stress disorder (PTSD) in children 6 years and younger and Scheeringa’s alternative PTSD algorithm (PTSD-AA) aim to be more developmentally sensitive for young children than the DSM-IV PTSD algorithm. However, very few studies compared the three algorithms simultaneously. The current study explores diagnostic outcomes of the three algorithms in young child survivors of accidental trauma.

**Methods:**

Parents of 98 young children (0–7 years) involved in an accident between 2006 and 2012 participated in a semi-structured telephone interview. Child posttraumatic stress symptoms (PTSS) were measured with the Anxiety Disorders Interview Schedule for DSM-IV-Child Version (ADIS-C/P), complemented with items from the Diagnostic Infant and Preschool Assessment (DIPA). Descriptive statistics were used to analyze the characteristics of the children, accident related information and PTS symptoms. We compared the three PTSD algorithms in order to explore the diagnostic outcomes.

**Results:**

A total of 9 of the children (9.2 %) showed substantial PTSS. Of these children 2 met the criteria of all three algorithms, 7 met both the DSM-5 subtype for children 6 years and younger and the PTSD-AA algorithm, and 2 did not fully meet any of the algorithms (subsyndromal PTSD).

**Conclusions:**

For young children, the DSM-5 subtype for children 6 years and younger and the PTSD-AA algorithm appear to be better suited than the previous DSM-IV algorithm. It remains important that clinicians pay attention to children with subsyndromal PTSD.

## Background

The Diagnostic and Statistical Manual of Mental Disorders, Fifth Edition (DSM-5) [1] includes a subtype for posttraumatic stress disorder (PTSD) in children 6 years and younger. Before the release of the DSM-5, several studies had shown that more developmentally sensitive PTSD criteria for young children were needed [2–4]. The PTSD criteria of the Diagnostic and Statistical Manual of Mental Disorders, Fourth Edition, Text Revision (DSM-IV-TR) [5] were based on research among adults and older children [2, 6]. Therefore, some of the symptoms were not suitable for young children, because they required skills that young children have not yet developed, such as verbal expression, memory or abstract thought [2, 7]. As a consequence, not all young children with substantial levels of posttraumatic stress symptoms (PTSS) did fully meet the required DSM-IV criteria for PTSD, although these children can experience impairment and need trauma-focused treatment [8].

In order to improve the identification of PTSD in young children, Scheeringa and colleagues, proposed alternative PTSD criteria for young children [4]. This alternative algorithm (PTSD-AA) focused on behavioral symptoms instead of thoughts and feelings, and included the following changes to the DSM-IV criteria. First, criterion A2 (response of fear, helplessness or horror) was removed because young children are less able to report their response to the traumatic event and witnesses are not always present. Second, the wording of some symptoms was adapted to make them more applicable for young children. Finally, the threshold to meet the avoidance/numbing criterion was lowered from 3 to 1 symptom [6]. These changes have been incorporated in the DSM-5 subtype for children 6 years and younger, in addition to the following (unrelated to the PTSD-AA proposal): First, criterion C avoidance/numbing has been split into *“Persistent avoidance of stimuli”* and *“Negative alterations in cognitions”.* Second, symptom C3 - “*Inability to recall an important aspect of the trauma”* and symptom C7 - *“Sense of a foreshortened future”* have been removed. Third, symptom C3 - *“Increased frequency of negative emotional states”* has been added to criterion C [1, 9]. In accordance with the PTSD-AA algorithm, criterion A2 was left out from the DSM-5. This criterion was considered redundant for the development of PTSD, because research showed that this criterion is common after experiencing a traumatic event and has little influence on the number of people who qualify for PTSD following a traumatic event [10]. In addition, other studies showed that people can develop PTSD without meeting criterion A2. For example, many professionals like military personnel or police officers do not have an emotional response to a traumatic event because of their professional training, but can still develop PTSD [11]. Table 1 presents an overview of the PTS symptoms and criteria of the DSM-IV, PTSD-AA algorithm and DSM-5 subtype for children 6 years and younger.

**Table 1:** Symptoms and Criteria of the Three Diagnostic Algorithms for PTSD

DSM-IV [5]	PTSD-AA [6]	DSM-5, subtype for children 6 years and younger [1]
Criterion A1	Criterion A1	Criterion A1
Criterion A2: Response to event involves intense fear, helplessness or horror	Criterion A2 not required	Criterion A2 not required
B. Intrusion (1 required)	B. Intrusion (1 required)	B. Intrusion (1 required)
1. Recurrent and intrusive distressing recollections	1. Recurrent and intrusive recollections, not required to be distressing	1. Recurrent and intrusive recollections, not required to be distressing
2. Recurrent distressing dreams of the event	2. Recurrent distressing dreams of the event	2. Recurrent distressing dreams of the event
3. Dissociation (*e.g.,* flashbacks)	3. Dissociation (*e.g.,* flashbacks)	3. Dissociation (*e.g.,* flashbacks)
4. Intense psychological distress at reminders	4. Intense psychological distress at reminders	4. Intense psychological distress at reminders
5. Physiological reactivity at reminders	5. Physiological reactivity at reminders	5. Physiological reactivity at reminders
C. Avoidance/numbing (3 required)	C. Avoidance/numbing (1 required)	C. Avoidance/negative alterations in cognitions and mood (1 required)
1. Efforts to avoid thoughts, feelings or conversations	1. Efforts to avoid thoughts, feelings or conversations	1. Efforts to avoid activities, places or physical reminders
2. Efforts to avoid activities, places or people	2. Efforts to avoid activities, places or people	2. Efforts to avoid people, conversations or interpersonal situations
3. Inability to recall an important aspect of the trauma	3. Inability to recall an important aspect of the trauma	3. Increased frequency of negative emotional states
4. Diminished interests in significant activities	4. Diminished interests, emphasize play constriction	4. Diminished interests, including play constriction
5. Feelings of detachment from others	5. Socially withdrawn behavior	5. Socially withdrawn behavior
6. Restricted range of affect	6. Restricted range of affect	6. Reduction in expression of positive emotions
7. Sense of foreshortened future	7. Sense of foreshortened future	
D. Hyperarousal (2 required)	D. Hyperarousal (2 required)	D. Hyperarousal (2 required)
1. Difficulty falling or staying asleep	1. Difficulty falling or staying asleep	1. Difficulty falling or staying asleep
2. Irritability, angry outbursts	2. Irritability, includes excessive temper	2. Irritability, angry outbursts, includes extreme temper tantrums
3. Difficulty concentrating	3. Difficulty concentrating	3. Difficulty concentrating
4. Hypervigilance	4. Hypervigilance	4. Hypervigilance
5. Exaggerated startle response	5. Exaggerated startle response	5. Exaggerated startle response

It is important to compare the three algorithms and to explore the diagnostic outcomes of the algorithms among young children [12]. However, research in this area is scarce. A study on PTSD in young children with burn injuries demonstrated a prevalence rate of 4.6 % with DSM-IV, 25.4 % with DSM-5 and 24.6 % with PTSD-AA at 1 month after the injury [13]. Meiser-Stedman, Smith, Glucksman, Yule, and Dalgleish [14] found a PTSD prevalence rate of 1.7 % with the DSM-IV algorithm and 10 % with the PTSD-AA algorithm in young children who were involved in a motor vehicle accident. Scheeringa, Myers, Putnam, and Zeanah [6] compared PTSD diagnoses according to the DSM-IV, PTSD-AA, DSM-5 and DSM-5-under consideration (DSM-5-UC) algorithm in children aged 3 to 6 years exposed to diverse types of trauma. They found that the percentage of children who qualified for a PTSD diagnosis was significantly lower when using the DSM-IV algorithm (13 %) compared to the PTSD-AA algorithm (45 %), DSM-5 algorithm (44 %) or DSM-5-UC algorithm (49 %). In order to understand PTSD in young children and prevent underdiagnosis, a broad research base is needed, including research in various countries and after various types of trauma exposure.

In the present study we compared the three main PTSD algorithms for young children age 0 to 7 years in an accidental injury sample involving various types of exposure (*e.g.,* road traffic accidents, near drowning, falls).

## Method

### Participants and procedure

The current study was part of a larger retrospective exploratory study and was conducted in the Academic Medical Center (AMC) and the VU University Medical Center (VUmc), both academic hospitals in Amsterdam, the Netherlands, with a level 1 trauma center. The study was approved by the Medical Ethics Committees of both hospitals.

All children age 0 to 7 years who had been involved in an accident, transported to the hospital by ambulance, and treated in the trauma (resuscitation) room between January 2006 and August 2011 were eligible for inclusion. Although the DSM-5 PTSD subtype for children 6 years and younger and the PTSD-AA algorithm are studied in children up to 6 years of age, we included 7-year-old children as well. In the clinical practice in the Netherlands, the distinction between age categories of children is often made as follows: young children are referred to as children aged 0–7 years and older children are referred to as children aged 8–17 years. In addition, many measures for PTSD use the same age categories. In order to stay close to the clinical practice and not to “forget” children aged 7 years, we decided to include 7-year-old children as well.

One child per family was included. Exclusion criteria were: living abroad, unknown place of residence and telephone number, permanent neurological injury and injured due to child abuse. Children who were injured due to child abuse were excluded because of hospital’s policy for this group of children. The policy implied that we could not contact these children for our study. In order to identify eligible children for the study we used the trauma registration system(s) of the Emergency Department.

First we performed a pilot study in order to test the procedures and measures. Thereafter, parents of the selected children received a letter in November 2011 containing information about the study. From December 2011 to February 2012, we contacted parents via telephone and invited them to participate. Informed consent was obtained from all participating parents. After informed consent, the first (MRG) and second author (EPMM) conducted a telephone interview with the parents.

### Measures

We constructed a semi-structured interview for parents whose child had been involved in an accident (Meynen M, van Meijel EPM, Gigengack MR, Lindauer RJL. Unpublished instrument), based on an existing protocol [15]. This protocol for health-care professionals contains examples of questions on children’s and parents’ acute and posttraumatic stress reactions to a traumatic event and can be used as a screening tool for PTSS in children and parents. The semi-structured interview consists of 12 open-ended questions on the following areas: the accident and the injuries, other traumatic experiences, medical/psychological history, peri- and posttraumatic reactions of the child and the parent, and coping. We constructed the interview in consultation with a child and adolescent psychiatrist (the fourth author; RJLL) and a clinical psychologist/psychotherapist, both experienced clinicians in the field of trauma.

During the semi-structured interview parents were asked about PTS symptoms of their child in the past. The questions consist of an open-ended question (*‘Did you notice any changes in your child’s behavior in the period following the accident?’)* followed by close-ended questions concerning examples of PTS symptoms (*e.g.,* ‘*Did your child have trouble sleeping since the accident?’* and *‘Did your child have nightmares or bad dreams about the accident?’*). These questions serve as a skip-out criterion. If parents reported one or more PTS symptoms for their child, we further assessed child PTSS with the PTSD module of the Dutch version of the Anxiety Disorders Interview Schedule for DSM-IV - Child Version (ADIS-C/P) [16, 17]. The ADIS-C/P is a semi-structured interview to assess anxiety disorders and comorbidity in children. Test-retest reliability and interrater reliability of the ADIS-C/P range from good to excellent [18, 19]. The 17 ADIS-C/P questions are based on the 17 PTS symptoms of the DSM-IV and formed the basis of our PTSD interview. However, these questions did not fully cover the PTS symptoms according to the PTSD-AA algorithm and the DSM-5 subtype for children 6 years and younger. In order to measure PTS symptoms according to all three algorithms, we complemented the ADIS-C/P questions with a number of PTSD questions (questions 37, 41, 42, 44, 47, 51, 52, 54, 55) from the 2009 version of the Diagnostic Infant and Preschool Assessment (DIPA; Scheeringa MS. Unpublished instrument). The DIPA is a semi-structured interview to assess symptoms of 12 DSM-IV disorders in children from late in their first year to 6 years of age [20]. Preliminary data on the reliability and the criterion validity show that the DIPA appears to be a reliable and valid measure [20]. The DIPA-questions were translated into Dutch by the first (MRG) and second author (EPMM) in consultation with a clinical psychologist/psychotherapist and a child and adolescent psychiatrist (the fourth author; RJLL).

The ADIS-C/P questions were complemented with DIPA questions in order to measure all symptoms of the PTSD-AA algorithm and DSM-5 subtype for young children. No DIPA questions were added to the *intrusion* cluster of the ADIS-C/P. In the cluster *avoidance/negative alterations in cognitions and mood* a number of DIPA questions were added. First, the ADIS-C/P question regarding symptom C1 - ‘*Recurrent and intrusive distressing recollections*’ - was expanded with the following DIPA question ‘*Does s/he try to avoid conversations that might remind him/her of the trauma?*’ *-* ‘*Does s/he try to avoid private thoughts or feelings that might remind him/her of the trauma?*’ (question 37). Furthermore, the following DIPA question was added: ‘*Since the trauma has s/he become more distant from family members and friends? I mean, s/he doesn’t want to show affection or maybe even be around people?*’ (question 44)*.* This question measures the adjusted symptom C5 - *‘Socially withdrawn behavior’* - of the PTSD-AA and DSM-5 subtype for young children. In order to measure symptom C6 - *‘Reduction in expression of positive emotions*’ - of the DSM-5 subtype for young children, the DIPA question ‘*Since the trauma, s/he doesn’t show as many happy emotions - like smiles or laughs - on his/her face, or doesn’t show them as strongly as s/he used to?*’ was added (question 41)*.* PTSD interviews were administered before the release of the DSM-5. The ADIS-C/P and the DIPA did not yet contain the new DSM-5 symptom C3 of the subtype for young children - ‘*Substantially increased frequency of negative emotional states (e.g., fear, guilt, sadness, shame, confusion)’*. This symptom was measured with the following question derived from the DIPA ‘*Is your child more sad, angry or upset since the accident?’* (question 42). In the PTSD-AA algorithm and the DSM-5 subtype for children 6 years and younger the hyperarousal symptom ‘*Irritability, outbursts of anger*’ includes extreme temper tantrums. Therefore, the following part of DIPA question 47 was added to the hyperarousal cluster of the ADIS-C/P: ‘*Has s/he developed extreme temper tantrums since the trauma?*’*.*

PTS symptoms were scored present or absent based on the frequency. Symptoms were scored present if they occurred a couple of times a month. Intensity of the symptoms was based on the reported impairment. If parents reported no impairment, then children did not fulfill the criteria for substantial PTSS. Impairment was measured with DIPA questions about impairment in the following domains: parental relationships, sibling relationships, daycare provider/teacher relationships, relationships with peers, ability to act appropriately outside home or daycare/school and measure of child’s distress (questions 56 to 61).

If parents reported child PTSS in the past, they were also asked if the symptoms were still present (results are not presented and are available on request). If parents reported other experienced traumatic events besides the accident, the PTS questions were administered separately for each of the events.

### Data analysis

We used IBM Statistical Product and Service Solutions (SPSS) 19 for all analyses. Descriptive statistics were used to analyze the characteristics of the children, accident related information and PTS symptoms. Differences between participants and non-participants were analyzed using the chi-square test and Mann–Whitney U tests. We compared the DSM-IV PTSD algorithm, the DSM-5 PTSD subtype for children 6 years and younger and the PTSD-AA algorithm in order to explore the diagnostic outcomes of the algorithms.

We defined substantial PTSS as 1) the child met the criteria of all PTSD clusters of any of the three algorithms and the parent reported impairment (threshold PTSD) or 2) the child did not fully meet the criteria of all PTSD clusters of any of the three algorithms, but met two of the clusters in any of the three algorithms and the parent reported impairment (subthreshold PTSD). Subthreshold PTSD is clinically significant, because people with subthreshold PTSD can experience impairment and may require treatment [21]. Our definition of subthreshold PTSD is supported by a study on definitions of subthreshold PTSD according to the DSM-5 PTSD algorithm [21]. The results of this study show that full symptoms in two or three of the PTSD clusters is the best fit for subthreshold PTSD. The authors recommend that future studies should use this definition of subthreshold PTSD [21].

## Results

### Sample characteristics

A total of 270 children and their parents were eligible to participate in the study. Of these families, 140 could not be contacted (telephone number was out of service or the telephone was not answered), 24 parents refused to participate and the interview with 8 parents could not be scheduled during the study period.

We included 98 parents (75 mothers and 23 fathers) of 98 children. Demographic characteristics of the children and accident related information are shown in Table 2. There were no significant differences between participants and non-participants in terms of gender (*χ*^2^ = 0.87, *p* = .35), age (U = 951.5, Z = −1.46, *p* = .14) and duration of admission (U = 678.0, Z = −0.54, *p* = .59).

**Table 2:** Demographic Child Characteristics and Accident Related Information (n = 98)

	N (%)	Median	Mean (SD)	Min-Max
Gender				
Male	67 (68.4)	--	--	--
Female	31 (31.6)	--	--	--
Child age during accident	--	3	3.1 (2.2)	0–7
Child age during interview	--	6	6.2 (2.7)	1–13
Time between accident and interview (in months)	--	35	36.3 (20.6)	4–69
Trauma type				
Road traffic accident	28 (28.6)	--	--	--
Fall	49 (50.0)	--	--	--
Other, including burns and near drowning	21 (21.4)	--	--	--
Total days in hospital including (P)ICU^a^	81 (81.0)	1	4.7 (9.5)	1–57
Total days on (P)ICU^a^	27 (27.0)	1	3.6 (4.3)	1–14

-- = not applicable

^a^(P)ICU: (Pediatric) Intensive Care Unit

### Posttraumatic stress symptoms

A total of 14 parents reported one or more PTS symptoms in their child in the past following the accident and completed the ADIS-C/P and DIPA questions. Of this group, 9 children (9.2 % of the total study population) showed substantial PTSS and impairment. These 9 children consisted of 7 boys and 2 girls. The age of the children with substantial PTSS ranged from 1 to 7 years at the time of the accident (median 6 years, mean 5.0 years, SD = 2.2), and was distributed as follows: 1 year (1 child), 2 years (1 child), 4 years (1 child), 6 years (4 children) and 7 years (2 children).

PTSD criterion and diagnosis frequencies measured with the three PTSD algorithms are presented in Table 3. Two of the 9 children with substantial PTSS met all three algorithms. Using the DSM-5 subtype for children 6 years and younger and the PTSD-AA algorithm 7 children with substantial PTSS were identified; 2 of these children met all three algorithms and 5 children met the DSM-5 subtype for children 6 years and younger and the PTSD-AA algorithm but did not meet the DSM-IV algorithm. The DSM-5 subtype for children 6 years and younger and PTSD-AA algorithm identified the same children. The 2 children who showed substantial PTSS and impairment but did not fully meet any of the algorithms, met the criteria of two PTSD clusters but lacked one or more symptoms in the third cluster. One of these children lacked symptoms in the cluster intrusion and one child lacked one symptom in the cluster hyperarousal.

**Table 3:** PTSD Criterion and Diagnosis Frequencies in Young Children with Substantial PTSS

PTSD criterion or diagnosis	N (n = 9)	% (n = 9)	Prevalence rate % (n = 98)
Intrusion	8	88	--
Avoidance/numbing (DSM-IV; 3 symptoms)	2	22	--
Avoidance/numbing (PTSD-AA; 1 symptom)	9	100	--
Avoidance/negative alterations cognitions and mood (DSM-5; 1 symptom)	9	100	--
Hyperarousal	8	88	--
DSM-IV diagnosis	2	22	2.0
PTSD-AA diagnosis	7	77	7.1
DSM-5 diagnosis	7	77	7.1
Substantial symptoms but no diagnosis^a^	2	22	2.0

-- = not applicable

^a^Children met at least two clusters of symptoms of PTSD according to any of the algorithms

## Discussion

This is one of the first studies comparing the three most prominent diagnostic algorithms for PTSD simultaneously in a substantial sample of young children exposed to accidental trauma. We found that 9.2 % of the young children developed substantial PTSS following an accident. This finding is in line with a previous study on the PTSD-AA algorithm following a motor vehicle accident (10 %) [14].

Our findings indicate that both the DSM-5 subtype for children 6 years and younger and the PTSD-AA algorithm appear to be more sensitive for young children than the DSM-IV algorithm. Using these two algorithms most of the children with substantial PTSS were identified (7 out of 9). In contrast, a minority of the children with substantial PTSS met the criteria of the DSM-IV algorithm (2 out of 9). The improved sensitivity of the PTSD criteria for young children seems a step forward, now that more young children suffering from substantial PTSS can be identified and thereby offered treatment. We believe it is important to maximize the sensitivity and to identify as many young children with substantial symptoms and impairment as possible, instead of not identifying young children who do have substantial PTSS and might need treatment.

Intrusion and hyperarousal symptoms were common, however, in accordance with other studies [13, 22], most of the children (7 out of 9) did not meet the DSM-IV threshold of the avoidance cluster (3 symptoms). With the lower threshold from the PTSD-AA and DSM-5 subtype for children 6 years and younger (1 avoidance symptom required instead of 3) all children met the criterion. Besides the lower threshold, the following adaptation of avoidance symptoms in the DSM-5 subtype for young children might have made this cluster better suited for young children: the wording of some symptoms has been made more appropriate for young children, 2 symptoms which were not applicable for young children have been removed, and 1 symptom better suited for young children has been added [1, 9].

Our findings indicate that the DSM-5 subtype for children 6 years and younger and the PTSD-AA algorithm identify the same children with substantial PTSS. On the one hand, this seems evident because the algorithms are mainly similar and incorporated roughly similar changes to the DSM-IV criteria. For example, in both algorithms the wording of some symptoms was adapted to make them more applicable for young children and the threshold to meet the avoidance criterion was lowered from 3 to 1 symptom [6]. On the other hand, the algorithms are not completely similar, because the DSM-5 subtype for children 6 years and younger was slightly more adapted by removing 2 avoidance symptoms and adding 1 new symptom to the avoidance cluster [1, 9]. Scheeringa, Myers, Putnam, and Zeanah [6] found that these adaptations had a limited effect on the prevalence of the avoidance criterion. The prevalence of the PTSD-AA avoidance criterion and the prevalence of this criterion according to the DSM-5 subtype for young children was almost equal [6]. This might explain why both algorithms identify the same children, despite a number of dissimilar avoidance symptoms.

The prevalence rate of PTSD more than tripled when the PTSD-AA algorithm or the DSM-5 subtype for children 6 years and younger algorithm (7.0 %) was used instead of the DSM-IV algorithm (2.0 %), although still 2 of the 9 children who experienced substantial PTSS and impairment did not fully meet the criteria of one of the three algorithms (2.0 %). Scheeringa, Zeanah, Myers, and Putnam [8] measured PTSD in young traumatized children at three time points and also found that, in particular at the last time point, more children were impaired but not diagnosed with PTSD. Angold, Costello, Farmer, Burns, and Erkanli [23] suggest to classify impaired but undiagnosed children into a *not otherwise specified* category of a disorder, in order to improve the identification of these children. We suggest to pay attention to this group of children. Clinicians should be aware that children with substantial PTSS who do not fully meet the criteria of any of the PTSD algorithms, can be very impaired and might need treatment.

### Limitations and strengths

This is an exploratory and retrospective study with a number of limitations. We interviewed parents 4 months to 5 years after the accident of their child. Parents’ recollections of the accident and their child’s posttraumatic stress symptoms may have become biased over time. For example, parents and children with physical or psychological symptoms and a long rehabilitation period, may have had more negative recollections than parents and children who recovered quickly. In addition, we administered the interviews via telephone. Telephone interviews are considered less valid than face-to-face interviews, because people would be less likely to disclose during telephone interviews due to the lack of face-to-face interaction [24]. This might have lead to an underreport of PTSS in our sample. On the contrary, studies in which telephone interviews are compared to face-to-face interviews showed that telephone interviews lead to similar results as face-to-face interviews. Both are valid methods to measure several psychiatric disorders, including PTSD [24, 25].

ADIS-C/P and DIPA questions were administered if parents reported one or more PTS symptoms on the initial questions of the semi-structured interview. Due to this method, it is possible that some children may have suffered from substantial PTSS but their parents failed to mention symptoms. As a consequence, the ADIS-C/P and DIPA questions would not have been administered in these parents. Nevertheless, this does not seem likely because the initial questions contained examples of PTS symptoms from all PTSD clusters. We expected parents of children with substantial PTSS to recognize a number of these examples.

The validation study of the DIPA has not yet been finished in the Netherlands. Hence, apart from the pilot study, the Dutch DIPA-questions have not been extensively validated. Besides, the study was conducted before the release of the DSM-5. For this reason the ADIS-C/P and the DIPA were not yet adjusted to the DSM-5 changes.

Our sample size and the number of children who qualify for a PTSD diagnosis are limited. As a consequence, a relatively small difference exists between the number of children who qualify for a DSM-IV diagnosis and the number of children who qualify for a diagnosis with the PTSD-AA algorithm and the DSM-5 subtype for children 6 years and younger. Nevertheless, especially from a clinical point of view, we believe that this difference is important, because all of the children who qualify for a diagnosis are impaired and might need treatment. Furthermore, because our sample consists of young children exposed to accidental trauma, caution should be taken in generalizing the results to children involved in other types of traumatic events. Our study should be replicated with a larger sample size and with children exposed to various types of traumatic events.

Strengths of the study are the focus of the under-researched population of (very) young children and the use of a combination of clinical interviews to measure (several variations of) PTSD diagnoses. Furthermore, research on the comparison of three diagnostic PTSD algorithms for young children is scarce. With this study we aimed to contribute to the knowledge on this topic and to expand the research base.

## Conclusions

Our results suggest that the DSM-5 subtype for PTSD in children 6 years and younger is an important improvement in identifying young children with PTSD compared to the DSM-IV algorithm. Nevertheless, clinicians should still be aware that some children with subsyndromal PTSD who may need trauma-focused treatment can stay unidentified.

## Abbreviations

PTSD: Posttraumatic stress disorder
PTSS: Posttraumatic stress symptoms
ADIS-C/P: Anxiety Disorders Interview Schedule for DSM-IV - Child Version
DIPA: Diagnostic Infant and Preschool Assessment
AMC: Academic Medical Center
VUmc: VU University Medical Center
SPSS: Statistical Product and Service Solutions
